# Achieving Elimination as a Public Health Problem for *Schistosoma mansoni* and *S. haematobium*: When Is Community-Wide Treatment Required?

**DOI:** 10.1093/infdis/jiz609

**Published:** 2019-12-12

**Authors:** Jaspreet Toor, David Rollinson, Hugo C Turner, Anouk Gouvras, Charles H King, Graham F Medley, T Déirdre Hollingsworth, Roy M Anderson

**Affiliations:** 1 Big Data Institute, Li Ka Shing Centre for Health Information and Discovery, University of Oxford, Oxford, UK; 2 Department of Life Sciences, Natural History Museum, London, UK; 3 Global Schistosomiasis Alliance, Department of Life Sciences, Natural History Museum, London, UK; 4 Oxford University Clinical Research Unit, Wellcome Trust Major Overseas Programme, Ho Chi Minh City, Vietnam; 5 Centre for Tropical Medicine and Global Health, Nuffield Department of Medicine, University of Oxford, Oxford, UK; 6 Center for Global Health and Diseases, Case Western Reserve University, Cleveland, Ohio, USA; 7 Centre for Mathematical Modelling of Infectious Disease, London School of Hygiene and Tropical Medicine, London, UK; 8 London Centre for Neglected Tropical Disease Research, Department of Infectious Disease Epidemiology, Imperial College London, London, UK; 9 Medical Research Council Centre for Global Infectious Disease Analysis, Department of Infectious Disease Epidemiology, School of Public Health, Imperial College London, London, UK; 10 The DeWorm3 Project, Natural History Museum, London, UK

**Keywords:** schistosomiasis, mass drug administration, school-based treatment, community-wide treatment, elimination as a public health problem

## Abstract

The World Health Organization (WHO) has set elimination as a public health problem (EPHP) as a goal for schistosomiasis. As the WHO treatment guidelines for schistosomiasis are currently under revision, we investigate whether school-based or community-wide treatment strategies are required for achieving the EPHP goal. In low- to moderate-transmission settings with good school enrolment, we find that school-based treatment is sufficient for achieving EPHP. However, community-wide treatment is projected to be necessary in certain high-transmission settings as well as settings with low school enrolment. Hence, the optimal treatment strategy depends on setting-specific factors such as the species present, prevalence prior to treatment, and the age profile of infection.

Schistosomiasis remains an endemic parasitic disease affecting approximately 220 million people around the world [1]. Following establishment of the neglected tropical disease (NTD) roadmap set by the World Health Organization (WHO), elimination as a public health problem (EPHP) was set as the 2025 goal for schistosomiasis, defined as reaching less than 1% prevalence of heavy-intensity infections in school-aged children (SAC; 5–14 years old) [2, 3]. For intestinal schistosomiasis caused by *Schistosoma mansoni*, heavy-intensity infections are defined as greater than 400 eggs per gram of feces and for urogenital schistosomiasis caused by *S. haematobium*, this is defined as over 50 eggs per 10 mL of urine [4]. Interruption of transmission (reducing the incidence of new infections to zero) is the end goal set by WHO for countries able to aim for this objective [2].

To achieve EPHP, the WHO has recommended treatment guidelines based on the prevalence in SAC prior to treatment [4, 5]. Current guidelines have focused on targeting SAC as they are most likely to be infected, but treatment of adults (≥ 15 years old) considered to be at risk has also been recommended in areas with higher prevalence [6]. This is important given morbidities in adults, such as female genital schistosomiasis and the link to HIV. Pediatric treatments are under development, which may enable the inclusion of pre-SAC within treatment programs [7, 8].

There is a limited supply and availability, particularly in Africa, of the treatment drug, praziquantel, used to treat infected individuals. Merck KGaA is currently the sole donor of praziquantel with 250 million tablets available annually, primarily for SAC [9]. Praziquantel is typically used in school-based (targeting SAC only) or community-wide (targeting both SAC and adults) mass drug administration (MDA) programs in which a proportion of the population is treated without diagnosis of infection. Given the limited supply of praziquantel, it is vital that the appropriate treatment strategy is used to prevent unnecessary treatment and to enable the efficient use of this valuable resource, allowing redeployment to those needing treatment. Additional praziquantel over that available in donations can be purchased but comes at a further cost for treatment programs.

The new NTD roadmap for 2021–2030 is currently under discussion, along with the WHO 2030 goals and treatment guidelines for schistosomiasis [10]. Through analysis of mathematical models on schistosomiasis transmission dynamics and control measures, we aim to provide guidance on the optimal treatment strategies required for achieving EPHP. Importantly, we highlight that the decision between adopting a school-based or community-wide treatment strategy to reach EPHP depends on the epidemiological setting, particularly the species present, prevalence prior to treatment, and age profile of infection.

## METHODS

**Figure 1.**
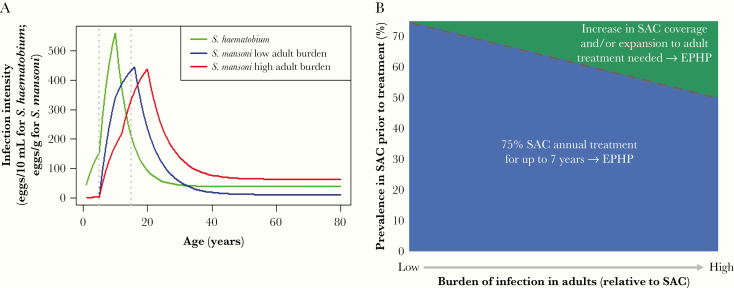
*A*, Age-intensity profiles of infection for *Schistosoma mansoni* using model-simulated low and high adult burdens of infection (relative to school-aged children [SAC; 5–14 years old]) and *S. haematobium* using previous fit to data [15]. *B*, Schematic showing treatment strategies required for achieving elimination as a public health problem (EPHP). Low adult burden of infection settings based on modeling insights on *S. mansoni* with a low adult burden setting and on *S. haematobium*. High adult burden of infection settings based on modeling insights on *S. mansoni* with a high adult burden setting. Blue region, 75% SAC-only annual treatment for up to 7 years is sufficient for achieving EPHP; green region, increase in school-based treatment coverage (ie, over 75% SAC annual treatment for 7 years) and/or expansion to community-wide treatment is needed for achieving EPHP (dashed line, approximate prevalence threshold above which this occurs for given age profiles).

Mathematical models of schistosome transmission and control have shown that the treatment strategy required to achieve EPHP will depend on the prevalence (transmission intensity) and age-intensity profile of infection. The age profile of infection varies as adults can harbor a low to high burden of infection corresponding to their exposure to infection relative to SAC (transmission intensity by age group; see Figure 1A and Supplementary Table 1).

Using the Imperial College London model (as previously described [11]), we investigated *S. mansoni* age profiles with low to high adult burden settings, and *S. haematobium* with a low burden in the adult population (as tends to be observed for *S. haematobium*; Figure 1A and Supplementary Data). For each age profile, we simulated low to high baseline prevalence settings and treated 75% of SAC only annually, for up to 7 years. In simulations where this strategy did not achieve EPHP, we increased SAC coverage and/or included adult treatment as needed. We assumed no migration (model simulations are for a single community of 500 individuals) and we assumed no acquired immunity.

## RESULTS

In low to moderate baseline prevalence settings (SAC prevalence <50% prior to treatment) for *S. mansoni*, analyses suggest that EPHP can be achieved by treating 75% SAC only annually for up to 3 years. In moderate-prevalence settings for *S. haematobium*, analyses suggest that EPHP can be achieved by treating 75% SAC only in 1 round of treatment. Note that in some low- to moderate-prevalence settings, the prevalence of heavy-intensity infections in SAC may already be under 1%, such that the EPHP goal is met prior to any treatment being carried out (Table 1). Despite achieving EPHP, a risk of resurgence remains if control efforts are not maintained.

**Table 1.** Model Recommended Treatment Strategies Required to Achieve EPHP in Low- to High-Prevalence Settings for *Schistosoma mansoni* and *S. haematobium*

Prevalence in SAC Prior to Treatment	Model Recommended Treatment Strategy
Low (<10%)	*S. mansoni*: 75% SAC annual treatment for 0–1 y (no treatment needed where EPHP met prior to treatment).
Moderate (10%–50%)	*S. mansoni*: 75% SAC annual treatment for 1–3 y (1–2 y for low adult burden of infection and 3 y for high adult burden of infection). *S. haematobium*: 75% SAC annual treatment for 0–1 y (no treatment needed where EPHP met prior to treatment).
High (≥50%)	*S. mansoni* and *S. haematobium* (where baseline SAC prevalence is 50%–51%): 75% SAC annual treatment for up to 1–4 y (1 y for *S. haematobium*; 2 y for low adult burden of infection, and 4 y for high adult burden of infection for *S. mansoni*).
	*S. mansoni* (where baseline SAC prevalence is below 73% and 59%, for low and high adult burdens of infection, respectively) and *S. haematobium* (where baseline SAC prevalence is below 70%): 75% SAC annual treatment for 7 y.
	*S. mansoni* and *S. haematobium* (with baseline SAC prevalences higher than those above): Increase in school-based treatment coverage (ie, over 75% SAC annual treatment for 7 y) and/or expansion to community-wide treatment needed. Coverage levels increase with the adult burden of infection.

Age-intensity profiles shown in Figure 1A were used.

Recommendations are for a single community (set at 500 individuals in the model). Corresponding parameter values, including prevalence threshold values for the age-intensity profiles investigated, are shown in Supplementary Tables 1–5.

Abbreviations: EPHP, elimination as a public health problem; SAC, school-aged children 5–14 years old; y, year(s).

In high baseline prevalence settings (SAC prevalence ≥50% prior to treatment), once the prevalence rises above a certain point, treatment of both SAC and adults becomes necessary [12, 13]. Importantly, adult treatment is not needed in all high-prevalence settings for achieving EPHP. The specific SAC prevalence threshold at which adult treatment is needed to reach EPHP varies with the age profile of infection. For *S. mansoni*, analyses suggest that treating 75% SAC only annually for up to 7 years is sufficient for achieving EPHP if the baseline SAC prevalence is below 73% or 59% for a low or high adult burden setting, respectively. For *S. haematobium*, similar to the low adult burden setting for *S. mansoni*, this holds for baseline SAC prevalence below 70%. For baseline SAC prevalence settings above these, intensified treatment is needed, such as higher coverage of SAC and/or an expansion in treatment coverage to include adults (Table 1 and Figure 1B; coverage levels required have been shown to increase with the adult burden of infection [12]).

To achieve EPHP, these modeling insights show that school-based treatment (of 75% SAC only) is sufficient in low- to moderate-prevalence settings for both *S. mansoni* and *S. haematobium* (Table 1). In certain high-prevalence settings, 75% SAC-only treatment remains sufficient for achieving EPHP; however, once the prevalence rises above a certain threshold, an increase in SAC treatment coverage and/or expansion to adult treatment is needed. This prevalence threshold decreases as the burden of infection in adults relative to SAC rises (Figure 1B). Overall, the optimal treatment strategy will depend on the setting, including factors such as the species present, prevalence prior to treatment, age profile of infection, and school enrolment levels.

Note that these insights derived from the predictions of mathematical models should not be overgeneralized to all settings. In addition to treatment coverage, individual adherence to treatment is also important. Our model assumes that 75% of SAC are treated at random in each round of MDA, hence SAC not adhering to treatment and SAC with no access to treatment (eg, schools with low enrolment) have not been considered. In settings with such challenges present, community-wide treatment may be more beneficial [14]. Additionally, the *S. haematobium* age profile of infection studied (informed by previous model fitting to data work [15]) has a low adult burden of infection but in areas with higher adult levels of infection, adult treatment will likely be needed.

### Caveats to These Analyses: The Skewed Distribution of Worms in Different Communities

**Figure 2.**
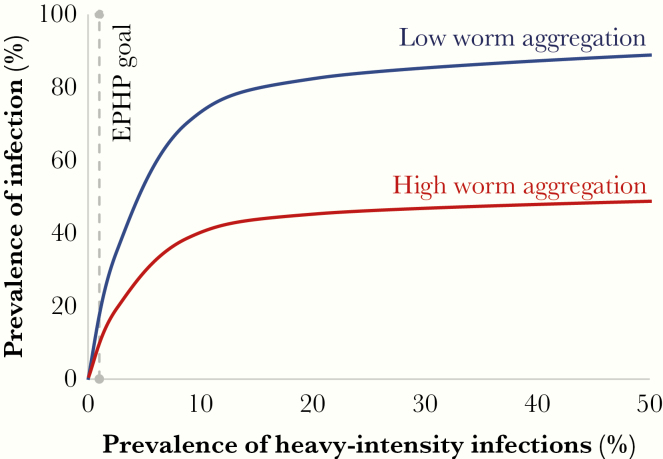
Schematic of nonlinear relationship between prevalence of infection and heavy-intensity infections prior to treatment for low (blue) and high (red) worm aggregation populations. Dashed line, prevalence of heavy-intensity infections is 1%, that is elimination as a public health problem (EPHP) is achieved for school-aged children 5–14 years old settings falling below this threshold.

Understanding the nonlinear relationship between prevalence of infection and heavy-intensity infections is key, particularly if the EPHP goal is changed to a goal that is based on prevalence of infection (Figure 2). This relationship varies with the degree of worm aggregation within a defined community (where a high worm aggregation corresponds to most individuals harboring zero or few worms and a few individuals harboring many) [11]. We know that the uneven distribution of worms across a community is common, but there are few measures of how variable it is in different places, and how it changes after treatment and during resurgence. Following multiple rounds of MDA, the degree of worm aggregation may increase, particularly if there are a few systematic nonadherers and/or nonaccess individuals remaining with heavy-intensity infections. Such individuals will reduce the potential impact of a treatment program and result in persisting infections, thereby increasing the risk of resurgence.

Low prevalence of heavy-intensity infections does not always correspond to low prevalence of infection (Figure 2). As a large reduction in intensity may be associated with a small reduction in prevalence, it is important that both prevalence and intensity data are collected to monitor the impact of a treatment program [11, 12, 14]. Even when the EPHP goal is achieved for *S. mansoni* and *S. haematobium* following treatment (ie, prevalence of heavy-intensity infections in SAC reduced to less than 1%), the prevalence of infection may still be high. This is due to light- to moderate-intensity infections persisting in SAC, in addition to light- to heavy-intensity infections remaining in pre-SAC and adults [13]. Hence, achieving EPHP may not equate to low levels of morbidity in non-SAC age groups and, furthermore, stopping treatment after achieving EPHP will likely lead to resurgence [16].

Alternative morbidity metrics have been proposed, such as prevalence of chronic and/or anatomic findings and quantifiable functional morbidities among SAC [17]. Further work is also needed for determining whether heavy-intensity infections in SAC is an informative indicator of adult morbidity. It is also important to consider the varying sensitivity of different diagnostic techniques when defining the goal. For example, due to its low sensitivity at low prevalence levels, a Kato-Katz prevalence measure is likely to be lower than a point-of-care circulating cathodic antigen prevalence measure [18–21].

## DISCUSSION

### School-Based Versus Community-Wide Treatment

The epidemiological setting influences whether school-based (SAC only) or community-wide (SAC and adult) treatment should be implemented. To prevent unnecessary treatment, targeting SAC only in low- to moderate-prevalence settings is sufficient for achieving EPHP. Community-wide treatment is not necessary in all high-prevalence settings. However, once a specific prevalence threshold is exceeded (determined by the epidemiological setting; Table 1), community-wide treatment becomes necessary. This threshold increases as the burden of infection in adults decreases (Figure 1B). As *S. haematobium* tends to have a low burden in adults, the threshold to necessitate community-wide MDA for this species is high (similar to *S. mansoni* with a low adult burden setting).

Due to the age profile of infection playing a key role in determining the optimal treatment strategy, it is vital that data are collected on the intensity of infection in each age group, specifically from SAC and adults in high-prevalence settings [12]. The data collected need to be representative of the age group, for example, sampling from only high-risk adults would overestimate the benefit of community-wide treatment [14]. Decisions of whether school or community-wide treatment are appropriate also need to consider the levels of school enrolment and treatment adherence within the area. With low levels of enrolment and SAC adherence, community-wide strategies may be more beneficial [14].

### Future Steps

As we move towards the 2030 WHO goals and treatment guidelines for schistosomiasis, it is vital that the optimal treatment strategies are recommended. This includes consideration of the epidemiological setting to determine whether school-based or community-wide treatment strategies are required for achieving EPHP. Community-wide treatment should be prioritized in settings where baseline prevalence is high (more specifically above a certain threshold determined by the age profile of infection) and where there is low school enrolment [12–14]. Ideally, rather than generalizing the treatment strategy by simply categorizing into low, moderate, and high baseline prevalence settings, treatment strategies should be determined based on key epidemiological factors in a given setting.

To prevent resurgence after achieving EPHP, programs will need to reassess their treatment strategies to either maintain EPHP or move towards interruption of transmission. With interruption as the goal, community-wide treatment is likely to be essential, alongside interventions such as improved water, sanitation, and hygiene (WASH) with behavior change [14, 22]. Currently, the ongoing Geshiyaro study is aiming to determine whether it is possible to achieve elimination with MDA alone in a region of Ethiopia with low *S. mansoni* prevalence.

As the epidemiological setting plays a key role in determining the optimal treatment strategy, mapping of areas is important. Development of mapping protocols (which capture key epidemiological parameters) will allow for more informed decisions to be made, thereby reducing overtreatment and allowing for treatment to be targeted to areas where it is needed. For example, sampling fewer SAC in more schools rather than many SAC in few schools has been found to increase the accuracy of prevalence estimates [23].

Universal health coverage, ensuring all those in need of treatment have equitable access to it, is a key objective for WHO. In addition to MDA, integration of treatment within local health systems would help ensure that treatment is available when needed [24]. More adult praziquantel donations are also needed. Furthermore, complementary interventions, such as WASH, behavior change, snail control, and a schistosome vaccine, could aid in the achievement of EPHP and interruption of transmission [25, 26]. Development of a pediatric formulation of praziquantel may also reduce transmission sooner, particularly in settings where pre-SAC harbor a high burden of infection.

In summary, to achieve EPHP, community-wide treatment needs to be prioritized in certain high-transmission settings (determined by the epidemiological setting, ie, factors such as the species present, prevalence prior to treatment, and age profile of infection) as well as settings with low school enrolment. However, in low- to moderate-transmission settings with good levels of school enrolment, school-based treatment is sufficient for achieving EPHP. By highlighting these insights, we hope to inform discussions on the schistosomiasis WHO treatment guidelines within the new roadmap for NTDs.

## Supplementary Data

Supplementary materials are available at *The Journal of Infectious Diseases* online. Consisting of data provided by the authors to benefit the reader, the posted materials are not copyedited and are the sole responsibility of the authors, so questions or comments should be addressed to the corresponding author.

jiz609_suppl_Supplementary_Data

## Notes

***Acknowledgments.*** We thank James E. Truscott for developing an initial version of the Imperial College London model code. We also thank the Global Schistosomiasis Alliance for facilitating important schistosomiasis meetings.

***Financial support.*** This work was supported by the Bill and Melinda Gates Foundation (grant number OPP1184344 to the Neglected Tropical Diseases Modelling Consortium, J. T., C. H. K., G. F. M., D. H., and R. M. A.); and the Wellcome Trust (grant number 089276/B/09/7 to H. C. T.).

***Supplement sponsorship.*** The authors acknowledge funding of the NTD Modelling Consortium by the Bill and Melinda Gates Foundation (OPP1184344).

***Potential conflicts of interest.*** All authors: No reported conflicts of interest. All authors have submitted the ICMJE Form for Disclosure of Potential Conflicts of Interest. Conflicts that the editors consider relevant to the content of the manuscript have been disclosed.
