# Supplementary material for: Achieving Elimination as a Public Health Problem for Schistosoma mansoni and S. haematobium: When Is Community-Wide Treatment Required?
Source: J Infect Dis. 2019 Dec 12;221(Suppl 5):S525–30. doi: 10.1093/infdis/jiz609 (PMC7289541; doi:10.1093/infdis/jiz609)
Supplement: jiz609_suppl_Supplementary_Data [file jiz609_suppl_supplementary_data.docx]

**Supplementary data**

**Model simulations**

In our simulations of preventive chemotherapy (PCT) impact on the control of schistosomiasis, we used a deterministic model developed by Imperial College London [[1](#_ENREF_1), [2](#_ENREF_2)]. We simulated low (SAC prevalence <10%), moderate (SAC prevalence 10-50%) and high (SAC prevalence ≥50%) baseline (i.e. prior to treatment) prevalence settings. The intrinsic intensity of transmission (basic reproductive number [R_0_]), was varied in the model to simulate this range of baseline prevalence levels. The age-specific contact rates, i.e. transmission intensities by age group, were varied such that the adult population had a low or high burden of infection relative to SAC (**Figure 1A**) [[3](#_ENREF_3), [4](#_ENREF_4)]. For *Schistosoma mansoni*, low and high adult burden of infection age profiles were investigated and for *S. haematobium*, an age profile (with a low adult burden of infection) from a previous model fit to data was investigated (**Table S1** and **Figure 1A**; [2]). The simulations were run for a single community with a population size set at 500.

Note: Low prevalence settings (SAC prevalence <10%) were only simulated for *S. mansoni* due to model stability for the given parameter values. Stable low prevalence settings were not produced for *S. haematobium*.

Beginning with an untreated population, we treated the population annually for up to 7 years (from year 0 to year 6 i.e. 7 rounds of treatment). Throughout the 7 years of annual treatment, we assumed PCT coverage of 75% of SAC-only (level of SAC receiving treatment; assumed to be delivered at random at each round within the SAC population). At year 7 (one year after the last round of treatment), the SAC heavy-intensity infection prevalence was evaluated to determine whether the WHO elimination as a public health problem (EPHP) goal had been met. Where the goal had not been achieved, we investigated the impact of increasing SAC coverage and/or expanding to include adult treatment.

Our models simulated what happened as annual treatment was carried out for various scenarios whilst projecting both the prevalence of infection (>0 eggs per gram [epg] for *S.* *mansoni* and >0 eggs/10ml for *S.* *haematobium*) and prevalence of heavy-intensity infections (≥400 epg for *S.* *mansoni* and ≥50 eggs/10ml for *S.* *haematobium* [[5](#_ENREF_5)]) in SAC throughout the treatment period. Note that some low prevalence settings had no heavy-intensity infections in SAC prior to treatment (but lower intensity infections were still present as SAC prevalence was not zero). See **Table S1** for parameter values used within the models.

The age profiles used within our analysis impact the results, hence the threshold at which adult treatment becomes necessary will vary depending on the epidemiological setting.

**Model code**

The Imperial College London deterministic model code has been made available by the NTD Modelling Consortium (ntdmodelling.org/diseases/schistosomiasis-mansoni). The results can be produced for this paper using the parameter values specified in **Table S1** and by setting the treatment frequency, coverage and length of treatment accordingly.

**Table S1:** Parameter values used for *Schistosoma mansoni* and *S. haematobium*.

| **Parameter** | **Value** | **Source** |
| --- | --- | --- |
| Fecundity | *S. mansoni*: 0.34 eggs/female/sample  *S. haematobium*: 0.3 | [[2](#_ENREF_2), [6-8](#_ENREF_6)] |
| Egg distribution within the individual | *S. mansoni*: 0.87  *S. haematobium*: 0.5 | [[2](#_ENREF_2), [7](#_ENREF_7), [8](#_ENREF_8)] |
| Aggregation parameter | 0.04 for low prevalence settings; 0.24 for high prevalence settings | [[1](#_ENREF_1), [2](#_ENREF_2), [9](#_ENREF_9)] |
| Density dependent fecundity | *S. mansoni*: 0.0007/female worm  *S. haematobium*: 0.0006/female worm | [[1](#_ENREF_1), [2](#_ENREF_2), [4](#_ENREF_4)] |
| Worm lifespan | *S. mansoni*: 5.7 years  *S. haematobium*: 4 years | [[2](#_ENREF_2), [6](#_ENREF_6), [10](#_ENREF_10)] |
| Drug efficacy | *S. mansoni*: 86.3%  *S. haematobium*: 94% | [[2](#_ENREF_2), [11](#_ENREF_11)] |
| *Mansoni* low baseline (prior to treatment) prevalence setting: Age specific contact rates for 0-4, 5-9, 10-15, 16+ years old | 0.032, 0.162, 1, 0.06 | [[1](#_ENREF_1), [12](#_ENREF_12)] |
| *Mansoni* low adult burden setting: Age specific contact rates for 0-4, 5-9, 10-15, 16+ years old | 0.01, 1.2, 1, 0.02 | [[3](#_ENREF_3), [4](#_ENREF_4)] |
| *Mansoni* high adult burden setting: Age specific contact rates for 0-4, 5-11, 12-19, 20+ years old | 0.01, 0.61, 1, 0.12 | [[3](#_ENREF_3), [4](#_ENREF_4)] |
| *Haematobium* age specific contact rates for 0-4, 5-9, 10+ years old | 0.3, 1, 0.02 | [[2](#_ENREF_2)] |
| Prevalence of infection | Percentage of population having > 0 eggs per gram [epg] (*S. mansoni*) or > 0 eggs/10ml (*S. haematobium*) | - |
| Heavy-intensity infection prevalence | Percentage of population having ≥ 400 epg (*S. mansoni*) or ≥ 50 eggs/10ml (*S. haematobium*) | [[5](#_ENREF_5), [13](#_ENREF_13)] |
| Human demography | Based on Uganda’s demographic profile | [[14](#_ENREF_14), [15](#_ENREF_15)] |

**Table S2: Low baseline prevalence setting for *S. mansoni*.** Levels of school-aged children (SAC; 5-14 years of age) and adult (≥ 15 years of age) coverage required to meet the WHO elimination as a public health problem (EPHP) goal using annual treatment. 0-year programme: EPHP goal is met prior to treatment.

| **Prevalence in SAC prior to treatment** | **Model recommended treatment strategy for achieving EPHP for *S. mansoni*** |
| --- | --- |
| Low (<10%)  Baseline prevalence in SAC: 8.9 – 9.7%  Baseline prevalence in adults: 9.2 – 10%  R_0_ values: 1.205 - 1.215  k values: 0.04 | 0- to 1-year programme  SAC: 75%  Adults: 0% |

**Table S3: Low burden setting in adults for *S. mansoni*.** Levels of school-aged children (SAC; 5-14 years of age) and adult (≥ 15 years of age) coverage required to meet the WHO elimination as a public health problem (EPHP) goal using annual treatment.

| **Prevalence in SAC prior to treatment** | **Model recommended treatment strategy for achieving EPHP for *S. mansoni*** |
| --- | --- |
| Moderate (10-50%)  Baseline prevalence in SAC: 12 – 49.8%  Baseline prevalence in adults: 7.6 – 32.7%  R_0_ values: 1.22 - 1.196  k values: 0.04 - 0.24 | 1- to 2-year programme  SAC: 75%  Adults: 0% |
| High (≥50%)  Baseline prevalence in SAC: 51.4 – 76.3%  Baseline prevalence in adults: 34.3 – 66%  R_0_ values: 1.198 - 3.0  k value: 0.24 | 2- to 7-year programme (Baseline prevalence below 72.6% SAC + 60.8% adults)  SAC: 75%  Adults: 0%  7-year programme (Baseline prevalence: 76.3% SAC + 66% adults)  SAC: 90%  Adults: 45% (or SAC 100% + adults 0%) |

**Table S4: High burden setting in adults for *S. mansoni*.** Levels of school-aged children (SAC; 5-14 years of age) and adult (≥ 15 years of age) coverage required to meet the WHO elimination as a public health problem (EPHP) goal using annual treatment.

| **Prevalence in SAC prior to treatment** | **Model recommended treatment strategy for achieving EPHP for *S. mansoni*** |
| --- | --- |
| Moderate (10-50%)  Baseline prevalence in SAC: 10.9 – 49.7%  Baseline prevalence in adults: 11.1 – 50.2%  R_0_ values: 1.245 - 1.23  k values: 0.04 - 0.24 | 3-year programme  SAC: 75%  Adults: 0% |
| High (≥50%)  Baseline prevalence in SAC: 51.3 - 76%  Baseline prevalence in adults: 51.8 – 76.2%  R_0_ values: 1.24 - 4.0  k value: 0.24 | 4- to 7-year programme (Baseline prevalence below 58.9% SAC + 59.3% adults)  SAC: 75%  Adults: 0%  7-year programme (Baseline prevalence: 76% SAC + 76.2% adults)  SAC: 95%  Adults: 85% |

**Table S5: *S. haematobium*.** Levels of school-aged children (SAC; 5-14 years of age) and adult (≥ 15 years of age) coverage required to meet the WHO elimination as a public health problem (EPHP) goal using annual treatment. NA: not achievable unless length of treatment programme is increased above 7 years. 0-year programme: EPHP goal is met prior to treatment.

| **Prevalence in SAC prior to treatment** | **Model recommended treatment strategy for achieving EPHP for *S. haematobium*** |
| --- | --- |
| Moderate (10-50%)  Baseline prevalence in SAC: 10.8 – 49.4%  Baseline prevalence in adults: 5.2 – 27.9%  R_0_ values: 1.203 - 1.184  k values: 0.04 - 0.24 | 0/1-year programme  SAC: 75%  Adults: 0% |
| High (≥50%)  Baseline prevalence in SAC: 50.4 – 75.9%  Baseline prevalence in adults: 29 – 63.3%  R_0_ values: 1.185 - 3.0  k value: 0.24 | 1- to 7-year programme (Baseline prevalence below 70.3% SAC + 55% adults)  SAC: 75%  Adults: 0%  7 -year programme (Baseline prevalence: 75.9% SAC + 63.3% adults)  SAC: NA  Adults: NA |

**References**

1. Anderson RM, Turner HC, Farrell SH, Truscott JE. Studies of the transmission dynamics, mathematical model development and the control of schistosome parasites by mass drug administration in human communities. Advances in parasitology **2016**; 94:199-246.

2. Truscott JE, Gurarie D, Alsallaq R, et al. A comparison of two mathematical models of the impact of mass drug administration on the transmission and control of schistosomiasis. Epidemics **2017**; 18:29-37.

3. Toor J, Turner HC, Truscott JE, et al. The design of schistosomiasis monitoring and evaluation programmes: The importance of collecting adult data to inform treatment strategies for Schistosoma mansoni. PLoS Negl Trop Dis **2018**; 12:e0006717.

4. Turner HC, Truscott JE, Bettis AA, et al. Evaluating the variation in the projected benefit of community-wide mass treatment for schistosomiasis: Implications for future economic evaluations. Parasit Vectors **2017**; 10:213.

5. Committee WHOE. Prevention and control of schistosomiasis and soil-transmitted helminthiasis. World Health Organ Tech Rep Ser **2002**; 912:i-vi, 1-57, back cover.

6. Anderson RM, May RM. Population dynamics of human helminth infections: control by chemotherapy. Nature **1982**; 297:557-63.

7. De Vlas SJ, Gryseels B, Van Oortmarssen GJ, Polderman AM, Habbema JD. A model for variations in single and repeated egg counts in Schistosoma mansoni infections. Parasitology **1992**; 104 ( Pt 3):451-60.

8. de Vlas SJ, Nagelkerke NJ, Habbema JD, van Oortmarssen GJ. Statistical models for estimating prevalence and incidence of parasitic diseases. Stat Methods Med Res **1993**; 2:3-21.

9. Chan MS, Guyatt HL, Bundy DA, Booth M, Fulford AJ, Medley GF. The development of an age structured model for schistosomiasis transmission dynamics and control and its validation for Schistosoma mansoni. Epidemiol Infect **1995**; 115:325-44.

10. Fulford AJ, Butterworth AE, Ouma JH, Sturrock RF. A statistical approach to schistosome population dynamics and estimation of the life-span of *Schistosoma mansoni* in man. Parasitology **1995**; 110 ( Pt 3):307-16.

11. Zwang J, Olliaro PL. Clinical efficacy and tolerability of praziquantel for intestinal and urinary schistosomiasis-a meta-analysis of comparative and non-comparative clinical trials. PLoS Negl Trop Dis **2014**; 8:e3286.

12. Toor J, Alsallaq R, Truscott JE, et al. Are We on Our Way to Achieving the 2020 Goals for Schistosomiasis Morbidity Control Using Current World Health Organization Guidelines? Clin Infect Dis **2018**; 66:S245-S52.

13. World Health Organization. Schistosomiasis: Progress report 2001-2011 and strategic plan 2012-2020, **2013**.

14. Anderson R, Truscott J, Hollingsworth TD. The coverage and frequency of mass drug administration required to eliminate persistent transmission of soil-transmitted helminths. Philos Trans R Soc Lond B Biol Sci **2014**; 369:20130435.

15. Pullan RL, Kabatereine NB, Quinnell RJ, Brooker S. Spatial and genetic epidemiology of hookworm in a rural community in Uganda. PLoS Negl Trop Dis **2010**; 4:e713.
